# Leptospirosis presenting in a woman with fulminant hepatic failure from Wilson's disease: a case report

**DOI:** 10.1186/1752-1947-4-256

**Published:** 2010-08-10

**Authors:** Emmanuel A Andreadis, Gerasimos D Agaliotis, George P Mousoulis

**Affiliations:** 1 3rd Department of Internal Medicine "Evangelismos" State General Hospital, Athens, Greece

## Abstract

**Introduction:**

We report an unusual case of Wilson's disease that was revealed by presentation of leptospirosis. The prompt detection of this potentially life-threatening disease highlights the importance of careful investigation. To the best of our knowledge, this is the first reported case of leptospirosis involving the development of fulminant liver failure due to Wilson's disease.

**Case presentation:**

A 17-year-old Caucasian woman presented with fever, rigors, vomiting and scleral jaundice. Following clinical and laboratory evaluation she was diagnosed with leptospirosis. After remission of this disease her condition inexplicably deteriorated. Further investigations revealed that she had Wilson's disease.

**Conclusions:**

The unexplained deterioration of hepatic function in a young person in remission from leptospirosis should alert the clinician to the presence of an underlying disorder, such as Wilson's disease, the early detection of which is crucial to the prognosis. The mechanism that initiates the development of Wilson's disease is not fully understood, but it is thought that an intercurrent illness, such as viral infection or drug toxicity, could be implicated. In our case, leptospirosis appeared to precipitate the deterioration of liver function in a patient with Wilson's disease, advancing our knowledge of this association. This original case report could have a broader clinical impact across medicine.

## Introduction

Leptospirosis is a zoonosis with protean manifestation caused by the spirochete, *Leptospira interrogans*. It is usually characterized by sudden onset of fever, rigors, myalgias and headache and is occasionally accompanied by nausea, vomiting and diarrhea. The disease course is generally mild to moderate and is seldom complicated by liver failure [1]. Wilson's disease is a rare cause of liver disorder, whose clinical manifestations range from increased levels of aminotransferase and bilirubin, decreased serum ceruloplasmin and detectable Kayser-Fleischer rings to fulminant hepatic failure (FHF). It can also present with neurologic, hematologic and renal dysfunction and affects mainly females between five and 40 years of age. This typical presentation represents only 50 percent of patients ultimately diagnosed with Wilson's disease [2]. To our knowledge, an association between leptospirosis and Wilson's disease has not been reported.

## Case presentation

**Figure 1:**
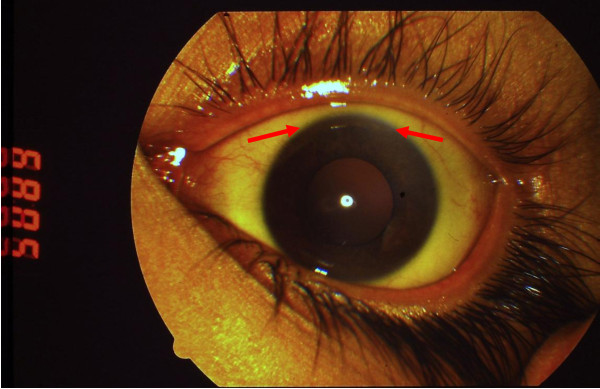
**Kayser-Fleisher ring in a 17-year-old woman with Wilson's disease**.

A 17-year-old Caucasian woman was admitted to the hospital following a seven-day history of malaise, with a temperature of 39°C, chills, anorexia, vomiting and scleral jaundice. Two days earlier she had discontinued treatment of norethisterone (Primolut-Nor), prescribed for polycystic ovaries. She was a resident of Athens, did not consume alcohol or take any illicit drugs and had not been exposed to rat excrement. Physical examination revealed a temperature of 38.8°C and mild epigastric tenderness on palpation without hepatosplenomegaly or mass. Apart from being jaundiced, there were no other signs of liver disease. Slurring of speech was evident on neurologic examination. There were no other clinical findings. White-cell count was 16,770/cm^3^, with 80% neutrophils; hemoglobin level was 9.4 g/dL, hematocrit 28.3%, with a normal mean corpuscular volume and 6% reticulocytes; platelet count was 240,000/cm^3 ^and erythrocyte sedimentation rates 36 mm/h. Analysis of the peripheral-blood smear showed a normal differential count with no band forms, basophilic stippling, or schistocytes. Coagulation profile: international normalized ratio (INR) 2.76, activated partial-thromboplastin time (aPTT) 72.11 s. Biochemistry findings showed 49 mg/dL glucose, 0.79 mg/dL serum creatinine, 2.8 g/dL albumin, 32 IU/L alkaline phosphatase (normal range 35 to 104), 33 IU/L alanine aminotransferase (normal range 5 to 40), 140 IU/L aspartate aminotransferase (normal range 5 to 37), 27.79 mg/dL total serum bilirubin (normal value < 1), 16.44 mg/dL direct bilirubin (normal value < 0.25), and 184 IU/L γ-glutamyltransferase (normal range 7 to 32). Tests for hepatitis C virus antibody (anti-HCV), hepatitis B (HBsAg, HBeAg, anti-HBc and anti-HBs), and hepatitis A antibody were negative, as were tests for antibodies against cytomegavirus (CMV), human immunodeficiency virus (HIV) types 1 and 2, anti-nuclear (ANA) and anti-mitochondrial (AMA) antibodies. A serum acetaminophen level was undetectable. Blood cultures obtained at admission were negative. Serologic test for *Leptospira interrogans *was positive (IgM > 1:80). Treatment was initiated with penicillin G 40,000 units/kg/day for seven days. Although our patient became afebrile, her mental status deteriorated and she displayed drowsiness and flapping tremor. Total serum bilirubin increased further to 66.7 mg/dL, INR reached 7.7 and ammonia (NH_3_) rose to 95.7 μmol/L (normal range 11 to 51). Hemolysis caused the hematocrit (Ht) level to drop to 17.6%. A Coombs' test was negative. No signs of bleeding were present. Lactulose and neomycin was administered and our patient received a transfusion of packed red blood cells, fresh frozen plasma and glucose. She was also given vitamin K but her coagulopathy remained unresponsive. Our patient appeared to have fulminant hepatic failure (FHF) with a Nazer score of eight in accordance with the prognostic index [3]. As the score was relatively high, she was referred to the National Transplant Organization for evaluation. It was decided that she was eligible for emergency liver transplantation and she was transferred abroad. However, within 20 days her clinical and biochemical condition showed signs of recovery and she was sent back to our department. A subsequent relapse prompted further investigation. Serologic test for *Leptospira interrogans *was repeated which showed lower levels of IgM antibodies (1/20). Having excluded the most common causes of acute liver failure, such as hepatitis A, B or drugs, it was reasonable to seek a less common etiology. In our young patient, the combination of neurological disorder, non autoimmune hemolytic anemia and unexplained liver disease along with negative Coombs, coagulopathy unresponsive to vitamin K, serum aminotransferases less than 2000 IU/L and normal or markedly subnormal alkaline phosphatase (< 40 IU/L), all suggested Wilson's disease. According to guidelines from the American Association for the Study of Liver Diseases (AASLD) patients in whom Wilson's disease is suspected should undergo screening with serum ceruloplasmin, 24-hour basal urinary copper, and slit-lamp examination for Kayser-Fleischer rings [1]. In our case, serum ceruloplasmin concentration was very low (7.0 mg/dL), and the 24-hour urinary copper excretion was 11,700 mcg (normal values ≤ 40 mcg). Slit-lamp examination detected Kayser-Fleischer rings (Figure 1). Given the deterioration in our patient's condition she was treated with a combination of zinc, which induces a negative copper balance by blocking intestinal absorption, and trientine, which acts as a potent copper chelator. Our patient showed gradual signs of improvement.

## Discussion

This case highlights the need for increased awareness in patients presenting with leptospirosis and liver disease, when the apparent remission of leptospirosis does not concur with improvement of liver function. The deterioration of our patient's clinical condition and the biochemical findings strongly point to an underlying disease that was not obvious at the initial presentation. Since other causes of FHF including viral, toxin or immunologic disease were excluded, the diagnosis of Wilson's disease underlying leptospirosis appeared more likely. The three most relevant features that characterize Wilson's disease include age < 35 years, Coombs negative hemolytic anemia and low serum alkaline phosphatase level, all of which applied to our patient. Diagnosis was established on the basis of Kayser-Fleischer rings, serum ceruloplasmin levels below 20 mg/dL and 24-hour urinary copper in excess of 40 mcg [4]. Massive release of copper from necrotic hepatocytes can display normal copper tissue concentration, as a result of which a biopsy sample could render false negative results. Having reached diagnosis in our case, liver biopsy proved unnecessary; our patient had FHF as defined by the development of acute hepatitis and encephalopathy in a person with no history of liver disease [5].

## Conclusions

The clinician should suspect an underlying disease in an unexplained liver failure associated with leptospirosis. The unexplained deterioration of hepatic function in a young person in remission from leptospirosis should alert the clinician to the presence of an underlying disorder, such as Wilson's disease, the early detection of which is crucial to the prognosis. As the literature has no documented reports of a leptospirosis infection being susceptible to severe liver disease, this case encourages further investigation.

## Consent

As the patient is a minor, written informed consent was obtained from her parents for publication of this case report and any accompanying images. A copy of the written consent is available for review by the Editor-in-Chief of the journal.

## Competing interests

The authors declare that they have no competing interests.

## Authors' contributions

EA was the attending physician and main author of the manuscript. GA assisted in the monitoring of the patient. GM contributed to the writing of the manuscript. All authors read and approved the final manuscript.
